# Multistable perception elicits compensatory alpha activity in older adults

**DOI:** 10.3389/fnagi.2023.1136124

**Published:** 2023-05-25

**Authors:** Kurtuluş Mert Küçük, Annika S. Wienke, Birgit Mathes, Canan Başar-Eroğlu

**Affiliations:** ^1^Department of Psychology, Izmir University of Economics, Izmir, Türkiye; ^2^Bremen Initiative to Foster Early Childhood Development (BRISE), University of Bremen, Bremen, Germany

**Keywords:** multistable perception, alpha oscillations, aging, bottom-up processes, event-related oscillations

## Abstract

Multistable stimuli lead to the perception of two or more alternative perceptual experiences that spontaneously reverse from one to the other. This property allows researchers to study perceptual processes that endogenously generate and integrate perceptual information. These endogenous processes appear to be slowed down around the age of 55 where participants report significantly lower perceptual reversals. This study aimed to identify neural correlates of this aging effect during multistable perception utilizing a multistable version of the stroboscopic alternative motion paradigm (SAM: endogenous task) and a control condition (exogenous task). Specifically, age-related differences in perceptual destabilization and maintenance processes were examined through alpha responses. Electroencephalography (EEG) of 12 older and 12 young adults were recorded during SAM and control tasks. Alpha band activity (8–14 Hz) was obtained by wavelet-transformation of the EEG signal and analyzed for each experimental condition. Endogenous reversals induced gradual decrease in posterior alpha activity in young adults which is a replication of previous studies’ findings. Alpha desynchronization was shifted to anterior areas and prevalent across the cortex except the occipital area for older adults. Alpha responses did not differ between the groups in the control condition. These findings point to recruitment of compensatory alpha networks for maintenance of endogenously generated percepts. Increased number of networks responsible for maintenance might have extended the neural satiation duration and led to decreased reversal rates in older adults.

## 1. Introduction

The brain, as an organ, is subject to drastic structural and functional changes across evolutionary timeline as well as during the lifespan of humans (Başar, 2011). However, there are quasi-invariant frequencies that make up the brain’s electrical activity that appear to be relatively constant. Alpha frequency is one of the most studied of such frequencies: it is observed in invertebrates (Schütt and Başar, 1992), cats (Başar-Eroglu et al., 1991), and humans alike (Babiloni et al., 2006); and subject to drastic changes during the lifespan of a human (Yordanova and Kolev, 1997; Klimesch, 1999). For instance, activity in traditional alpha band (8–14 Hz) is not observed in children until about the age of 3 (Başar, 2011). However, it becomes the predominant resting rhythm at occipital area at around 18 years of age. This is also subject to change after the age of 55, where the resting alpha activity (Knyazeva et al., 2018) as well as evoked alpha (Kolev et al., 2002) spread through the cortex including anterior areas. We argue that these age-related changes are also relevant to multistable perception where researchers repeatedly observed decrease in perceptual reversals after the age of 55 (Jalavisto, 1964; Aydin et al., 2013; Kondo and Kochiyama, 2018).

Age-related alterations in the brain can influence multiple processes that might affect the rate of perceptual reversals in multistable perception. This measure is sensitive to various changes in bottom-up and top-down processes (Leopold and Logothetis, 1999; Kornmeier and Bach, 2012) and combination of these can have additive effects on the rate of reversals (Kornmeier et al., 2009). For instance, dwell times (i.e., duration of perceptual alternatives) can be shortened by prolonged adaptation of sensory neurons to that percept (Long and Toppino, 2004), which occurs naturally during long observation periods. This process could have been altered by the increase in neural noise (Voytek et al., 2015) and changes in adaptation of neural groups in older brains (Schmolesky et al., 2000), especially at posterior areas. A previous study reported that attending to visual cues of non-dominant interpretation reduced the duration of dominant interpretation (Mathes et al., 2006). Authors argued that attentional effort facilitated perceptual reversals by increasing the strength of neural signals representing the non-dominant alternative. This could also be relevant because older adults show impaired ability to maintain the perception of ongoing alternative via attentional effort (Aydin et al., 2013). Duration of dwell times can also be manipulated by disrupting the conflict resolution between bottom-up and top-down perceptual representations by inhibiting posterior parietal activity (Kanai et al., 2010, 2011). Authors of these studies argued that parietal shrinkage in older adults might influence the same process and lead to lower reversal rates. Given the multitude of functional and structural changes in the aging brain, it is not plausible to isolate just one factor as the driver of age-related decrease in reversal rates. As previous studies suggested, spatiotemporal integration of sensory information (Kondo and Kochiyama, 2018), attentional modulation of sensory areas (Aydin et al., 2013), and neural adaptation and noise in early sensory areas (Díaz-Santos et al., 2017; Arani et al., 2018) are all considered to contribute to decreased number of reversals and/or prolonged dominance durations in older adults.

**Figure 1:**
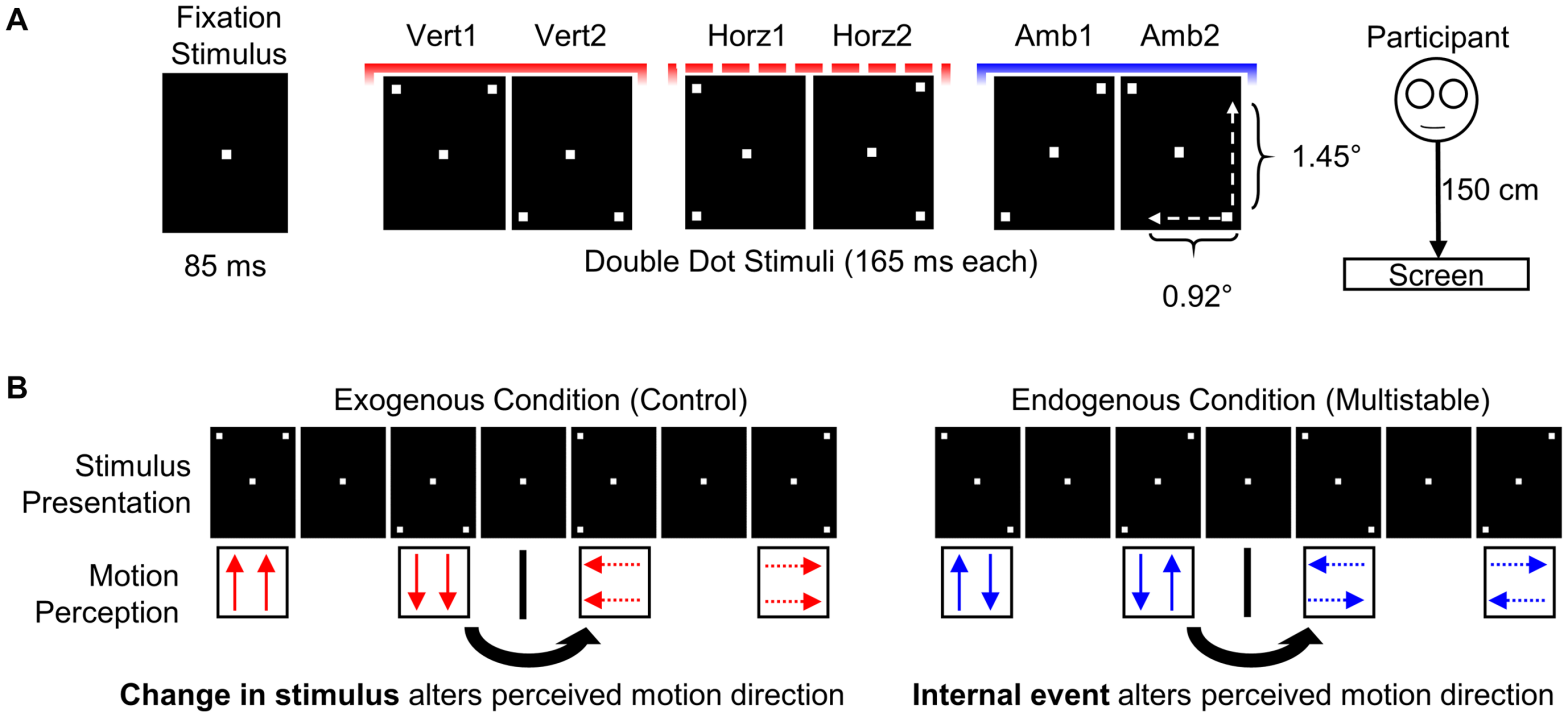
Schematic illustration of stimuli and tasks. **(A)** Stimulus properties. Stimulus duration was 165 ms for rectangles with two dots on corners (i.e., double dot stimuli) and 85 ms for fixation stimulus. Perception of motion direction was induced by repeated sequence of fixation, double dot, fixation, and double-dot stimuli of either vertical, horizontal, or multistable stimulus pairs. Viewing distance was 150 cm. **(B)** Sequence of stimulus presentation and change in perceived motion for exogenous (left) and endogenous tasks (right). Perceived exogenous vertical motion is generated by sequence of Vert1 and Vert2 stimuli and it changes to horizontal motion when sequence is changed to Horz1 and Horz2 stimuli. Onset of Horz1 marks the onset of *exogenous reversal* in this example. Multistable direction of motion is induced by sequences of Amb1 and Amb2 stimuli. Change in perceived motion occurs spontaneously even though display sequence remains the same. This is called as *endogenous reversal* (retrieved and modified from: Mathes et al., 2016).

Our aim is to narrow down the effect of aging via neural adaptation processes during multistable perception. Therefore, alpha response preceding endogenous (i.e., multistable) perceptual reversals was investigated in young and older adults. This alpha response represents the adaptation and destabilization of neural populations that are involved in maintaining the perceptual alternative in consciousness (İşoğlu-Alkaç et al., 2000; Strüber and Herrmann, 2002). To measure reversal related activity, we have employed the stroboscopic alternative motion stimulus (SAM) and a control stimulus (Figure 1, see Methods and Materials for details). Presenting both stimuli allows the comparison of perceptual reversal-related activity to other activities related to attention and decision making. The control stimulus also provides behavioral measures of reaction time and accuracy which is not possible with SAM. Given the increased noise (Voytek et al., 2015) and changes in neural adaptation in occipital cortex of older human and non-human animals (Schmolesky et al., 2000; Ding et al., 2017), we expected to observe an attenuation of the occipital alpha response in older adults during endogenous reversals. We also expected a spread in alpha activity towards anterior areas to compensate for this functional decrement in older adults, as was previously shown in simple sensory stimulation studies (Kolev et al., 2002). Furthermore, the topographical shift of alpha is not expected during exogenous reversals (i.e., control condition) in older adults. In other words, we expected to observe comparable occipital alpha responses in young and older adults during exogenous perceptual reversals. Because (i) different motion directions in control condition is provided externally, this eliminates the effect of neural adaptation on perceptual awareness and (ii) the change in stimulus itself leads to stronger top-down responses which would decrease the necessity for compensatory alpha activity (Schmiedt-Fehr et al., 2009).

## 2. Methods and materials

### 2.1. Participants

A total of 30 participants (15 young, 15 older) volunteered to participate in the study. Three of the older participants were excluded due to poor data quality. One young participant was also excluded as an outlier. To obtain equal sample sizes across groups, two young participants were pseudo-randomly selected and excluded from analyses. Finally, data of 12 young (3 male) and 12 older (2 male) participants were analyzed. All participants had normal or corrected to normal vision. Age groups were matched in years of education they received (Table 1). There was one left-handed participant in each group. Participants with head trauma, psychiatric diagnoses, or psychiatric medicine use were not included in the study. This study was approved by ethics committee in Bremen University and all participants signed an informed consent form before participating in the study.

**Table 1:** Summary of participant demographics.

Demographics	Young adults	Older adults
*N* (male)	12 (3)	12 (2)
Mean Age (SD)	24.33 (2.43) years	62.17 (3.93) years
Handedness	11 right/1 left	11 right/1 left
Education (SD)	14.37 (2.01) years	15.33 (2.71) years

### 2.2. Stimuli

A black rectangle on a white background was centered on the computer screen. Three different sequences of stimuli were created using this base rectangle: one for endogenous multistable motion, one for vertical exogenous motion, and one for horizontal exogenous motion (Figure 1). Stroboscopic alternative motion (SAM) was created by subsequent repeated presentation of diagonal double-dot and fixation stimuli (see Supplementary Video S1). Sequence of stimulus presentation was in the following order and repeated throughout the experiment: fixation, double-dot, fixation, double-dot. Duration of each such sequence was approximately 500 ms. A slightly modified version of the SAM rectangles was used to create the control stimulus (i.e, exogenous stimulus). Double-dot stimuli were placed at either horizontal or vertical corners instead of diagonal corners for this version (Figure 1). Motion change was externally applied in exogenous stimulus display by changing the double dot stimulus sequence (see Supplementary Video S2). A computer controlled 19″ CRT (Cathode-Ray Tube) monitor was used for stimulus presentation.

### 2.3. Stroboscopic motion tasks

Schematic illustration of both conditions and each of the perceived motions are presented in Figure 1. The endogenous condition employed the stroboscopic alternative motion stimulus (SAM). Participants reported internally generated spontaneous reversals of the direction in motion perception by pressing a button on a custom response device. There are two visual illusions at play in this task: (1) one is the illusion of stroboscopic motion where participants perceive dots moving from one location to another and (2) the illusory alternation of the motion direction due to the diagonal positioning of the dots (Mathes et al., 2016). Participants either experience vertical or horizontal motion during continuous observation.

The exogenous condition was utilized as a control measure for the endogenous condition to ensure that participants correctly responded with a button press when perceiving a reversal of motion direction. The alteration in task design to the classical version of SAM allowed the assessment of reaction times upon externally (i.e., by stimulus change) triggered motion reversals (Başar-Eroglu et al., 2016; Mathes et al., 2016). Similar to SAM, perceived stroboscopic motion of the exogenous stimulus was either vertical or horizontal. Number of external reversals within a minute was set to eight for the exogenous stimulus in both groups; a slightly lower number compared to previous studies in younger adults (Mathes et al., 2016). This modification was undertaken due to lower reversal rates of older adults (Jalavisto, 1964) and, thus, achieve a comparable number of reversals during the endogenous and the control condition.

### 2.4. Procedure and EEG recording

Participants were seated in a dimly lit, sound proof, electromagnetically shielded room. Experimenters informed participants about the endogenous SAM reversals and explained that they have to press the button whenever they experience a reversal. Participants were asked to focus to the central white dot of the stimulus throughout the experiment. Participants were trained for approximately 100 s to ensure that they understood the requirements of the two tasks. Duration of each task was 10 min, summing up to a total of 20 min.

The EEG was recorded using Ag-AgCl electrodes at F3, F4, C3, C4, P3, P4, T5, T6, O1, and O2 locations according to the 10–20 system. Earlobe electrodes were used as reference electrodes. Electrode impedances were below 5 kΩ for all electrodes. EOG was recorded from medial-upper and lateral-orbital rim of the right eye. The EEG was amplified with a Nihon Kohden (EEG-4421 G) EEG device with band limits 0.1–70 Hz. The EEG was digitized online with a sampling rate of 500 Hz. Digitized EEG was stored within a hard disc of a computer for offline EEG analysis. A 50 Hz Notch filter was applied to remove the mains interference. For the recording of EOG, the time constant 0.3 s with a low pass filter at 70 Hz was applied. All channels were displayed by a computer monitor to observe EEG activity during the recording.

### 2.5. Definition of epochs and artifact rejection

#### 2.5.1. Definition of epochs containing a perceptual reversal (reversal epochs)

**Figure 2:**
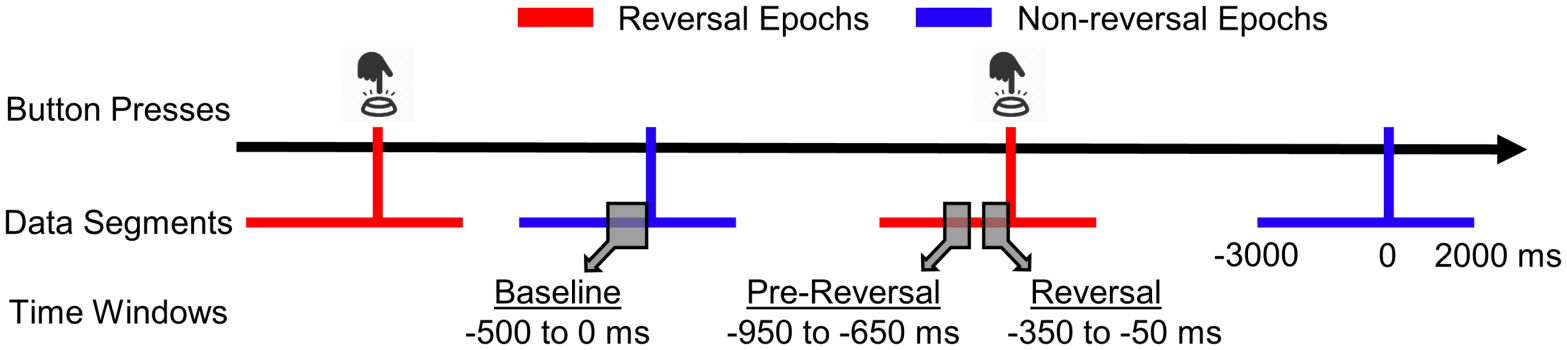
Depiction of data segmentation. Button presses are indicated with small button icons on short red bars. 3,000 ms before and 2,000 ms after the button presses were segmented for reversal epochs. Non-reversal epochs had the same duration but did not include button presses or time points from other non-reversal epochs. Baseline, pre-reversal, and reversal time windows (for details see Definition of Time Windows for Analyses) were used for averaging alpha activity (retrieved and modified from: Mathes et al., 2016).

Segmentation procedure is outlined in Figure 2. Reversal epochs were segmented within the time window ranging from 3,000 ms before to 2,000 after the button presses reporting a perceptual reversal (Başar-Eroglu et al., 2016; Mathes et al., 2016; Rürup et al., 2020). Segmentations that contain more than one button presses were excluded. This segmentation procedure was utilized to conduct *response locked averaging* for the epochs of both the endogenous and the exogenous conditions.

#### 2.5.2. Definition of epochs with no perceptual reversal (non-reversal epochs)

Onset of each double-dot display of SAM and exogenous stimulus was used for creating artificial markers on continuous EEG signal. This resulted in stimulus markers with 250 ms intervals throughout the continuous EEG session which were then utilized to extract epochs with 5 s duration that do not include button presses. Epochs that overlap with reversal epochs or with another non-reversal epoch were excluded. This resulted in *non-reversal epochs* that do not contain perceptual reversals. Therefore, these epochs represented perceptual stability periods.

Segmentation procedure resulted in four different set of epochs: endogenous response-locked reversal epochs, exogenous response-locked reversal epochs, endogenous non-reversal epochs, exogenous non-reversal epochs.

#### 2.5.3. Artifact rejection

Epochs containing multiple button presses were excluded to prevent the effects of post-motor activity. Visual inspection of each trial in unstable epochs of all tasks provided the following exclusion procedure for the time window of −1,500 ms to 200 ms around the button press demarcation: All epochs with eye movement or blink artifacts were manually excluded from analyses. Large drifts and spikes (±100μV) that could result from muscle activity, sweating, ocular artifacts, and changes in conductance were excluded from analyses. Artifact rejection was followed by random epoch exclusion procedure that ensured both groups had an average of 29.4 epochs (*SD* = 2.28) for each task and reversal condition.

### 2.6. Time-frequency decomposition

Time-frequency decomposition of the EEG data was conducted via wavelet convolution in frequency domain (for details see: Cohen and Donner, 2013) utilizing custom scripts in Matlab (2018) (The MathWorks). For each participant, EEG epochs of 5 s for all conditions (reversal and non-reversal epochs) were concatenated prior to wavelet transformation and segmented again thereafter in order to reduce wavelet folding artefacts. Selected electrodes were F3, F4, C3, C4, P3, P4, O1, and O2. Then complex wavelets were constructed and scaled for each frequency by:


(1) ψ(t)=(e2πift)(e−t22σ2)

(2) $$\sigma =\frac{n}{2\pi f}$$

Where *t* is time, *f* is frequency, $\sigma^{2}$ scales the gaussian window of the complex wavelets according to *n,* which is the number of wavelet cycles. Frequencies ranged from 8 to 14 Hz in 0.25 steps. Number of wavelet cycles was *n* = 7. Complex wavelets were normalized to have unit energy. The concatenated signals and the complex wavelets were then transformed into frequency domain using the built-in Fast Fourier transform algorithm (*fft.m* function in MATLAB; Frigo and Johnson, 1998) in Matlab (2018). Fast Fourier transform (FFT) is defined (Eq. 3) for vector *x* and *n* sampling points by:


(3) $$y_{k}=\overset{n}{\underset{j=1}{\sum }}\left(\omega_{n}^{\left(j-1\right)\left(k-1\right)}x_{j}\right)$$

Where ω=e−2πin is one of n complex roots of unity and *i* is the imaginary unit. Resulting power spectrums of the signal and the complex wavelets were then multiplied. Result of this multiplication was transformed into time domain using the built-in inverse FFT (iFFT) algorithm in Matlab (2018). Using the same definitions above, with vector *x, n* sampling points, and ω; iFFT is defined by:


(4) $$x_{j}=\overset{n}{\underset{k=1}{\sum }}\left(\omega_{n}^{\left(j-1\right)\left(k-1\right)}y_{k}\right)$$

The resulting complex signal was then reshaped into a format where conditions, trials, and time points are separated, identical to the signal format before concatenation. Then frequency-band specific power *A* was calculated for each participant at each time point, and averaged over trials in each condition by:


(5) $$A\left(t\right)=\overset{n}{\underset{k=1}{\sum }}{\left|Z{\left(t\right)}_{k}\right|}^{2}$$

Where Z is the complex signal, *t* is time, and *n* is the total number of trials of a participant in a given condition.

To illustrate the changes in oscillatory activity in reversal epochs relative to non-reversal epochs, baseline normalization was conducted separately for each participant, each task, and channel. In accordance with previous studies, it was decided to take averaged values of non-reversal epochs as the baseline activity instead of assessing remote time points within the unstable epochs (Mathes et al., 2014; Başar-Eroglu et al., 2016; Mathes et al., 2016). Baseline windows started 500 ms before the epoch marker (0 ms) and ended at the zero-time point. Power values within the baseline time window were averaged across time points for each frequency, resulting in one mean value for each frequency (Cohen, 2014). Each of the baseline values were then log transformed and subtracted from each log transformed spectral estimate of averaged unstable epochs for each task and participant (Eq. 6). This procedure created baseline normalized time-frequency power values for each participant and task. Relative change in activity in unstable epochs was represented in decibels (dB):


(6) $$dB=10\times \log_{10}\left(power/baseline\right)$$

Normalized time-frequency power values were then averaged within the specified time windows for alpha band and used in statistical analyses. The center frequency for the alpha band was set to 11 Hz for all participants, and 95% of its activity included the activity between 7.87 and 14.13 Hz (see Supplementary Figure S1). This range was defined as two standard deviations of the wavelet in the frequency domain. Given that wavelet cycle was set to 7, current center frequency (11 Hz) was the only frequency in the alpha band range that includes all of the activity in traditional alpha band (8–14 Hz).

### 2.7. Definition of time windows for analyses

Time windows were defined according to the button press demarcation in both the exogenous and the endogenous tasks. Therefore, time intervals before the button presses are shown by minus “−” symbol to indicate their relative time point to the button press. Two time windows were defined for endogenous condition: pre-reversal (−950 to −650 ms) and reversal (−350 to −50 ms) windows. Pre-reversal period represented the activity before the perceptual reversal. This window was contrasted to reversal window so that the reversal related decrease in alpha activity could be shown. Time windows for the exogenous task were defined by sliding pre-reversal (−850 to −550 ms) and reversal (−250 to 50 ms) windows 100 ms towards the button press to account for slight time shift of oscillatory responses between the conditions (Başar-Eroglu et al., 2016; Mathes et al., 2016). A third time window, *baseline window*, representing the baseline activity was extracted from the 500 ms interval preceding the artificial marker of non-reversal epochs. Activity in pre-reversal and reversal windows represented reversal-related change in alpha power relative to baseline window in decibel units (dB); whereas activity in the baseline window reflected the alpha power (μV^2^) during perceptual stability without baseline correction. The wavelet’s width in time was estimated as the twice of its folding time exp (−2) and found to be approximately 300 ms for 11 Hz center frequency (Torrence and Compo, 1998). Each of the time intervals interjecting those three time windows of interest were longer than 150 ms, indicating that separation of the time windows were adequate.

### 2.8. Data analysis

#### 2.8.1. Behavioral analyses

IBM SPSS Statistics 25 was utilized for behavioral analyses. Endogenous reversal rates were calculated by dividing the number of reversals by the number of minutes in the endogenous condition. Reversal rates were compared between age groups with an independent samples t-test and Cohen’s *d* was reported for effect size. Reaction time (RT), RT variability, and accuracy scores (percentage of correct responses) were compared between the age groups with the behavioral data obtained during the exogenous condition. Homogeneity of variances were violated both for reaction time (*F* [1, 22] = 8.915, *p* = 0.007) and accuracy scores (*F* [1, 22] = 8.131, *p* = 0.009). Therefore, Mann–Whitney U tests were employed to compare reaction times and accuracy scores between young and older participants.

#### 2.8.2. Alpha time-frequency power

IBM SPSS 25 was utilized for analyzing alpha activity. Analyses were conducted on averaged electrode pairs at frontal (mean: F3, F4), central (mean: C3, C4), parietal (mean: P3, P4), and occipital (mean: O1, O2) locations. The averaged signals for each region of interest (ROI) are abbreviated as F, C, P, and O, respectively. Baseline normalized alpha time-frequency power (i.e., alpha enhancement/suppression) was computed for the pre-reversal and reversal time windows of reversal epochs. 11 Hz event-related spectral perturbations were analyzed with a three-way mixed ANOVA with age group (2 levels: young, older) as the between subjects factor, ROI (4 levels: F, C, P, O), and time (2 levels: pre-reversal, reversal) as the within subjects factors. This analysis was conducted separately for endogenous and exogenous conditions. Partial eta squared was reported as the effect size of significant main, interaction, and contrast effects. All repeated measures effects were reported assessing Greenhouse–Geisser correction to correct for violations of sphericity as indicated by Mauchly’s Sphericity test. Post-hoc comparisons following significant main or interaction effects were corrected using the Bonferroni procedure.

Planned paired samples t-tests were conducted to investigate reversal-related change in alpha power from pre-reversal to reversal window for each group, ROI, and condition, separately. Hedge’s *g_av_* was reported as effect size to correct for small sample size and paired comparisons (Lakens, 2013). These comparisons were important as they can show involvement of different alpha networks at different locations for each group and task.

Differences in alpha power in stable epochs were analyzed with two mixed ANOVAs with one between-subjects factor age group (2 levels: young, older) and one within subjects factor ROI (2 levels: F, C, P, O) for endogenous and exogenous conditions, separately. These were conducted to investigate whether there were group differences in baseline alpha activity that can influence the analysis of relative alpha activity (dB).

## 3. Results

### 3.1. Behavioral results

**Figure 3:**
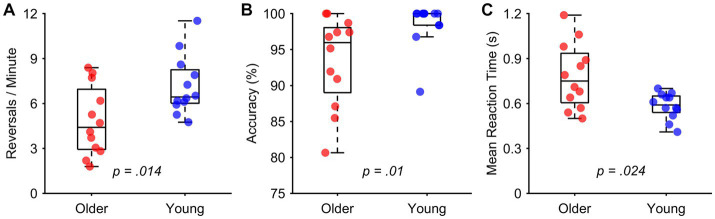
Results of behavioral comparisons. **(A)** Older adults had significantly slower reversal rates compared to young adults in endogenous condition. **(B)** Older adults were significantly less accurate than young adults while reporting the new motion direction of exogenous stimuli. **(C)** Older adults had slower RTs compared to young adults in exogenous condition.

Group comparisons of behavioral measures and their significance levels are depicted in Figure 3. Young participants showed significantly faster reversal rates (*M* = 7.18 ± 1.97) compared to older adults (*M* = 4.83 ± 2.26) during the endogenous condition *t* (22) = −2.683, *p* = 0.014, *d* = 0.97. Young participants responded significantly faster to changes in motion (*M* = 0.58 ± 0.09) compared to older participants (*M* = 0.78 ± 0.22) during the exogenous condition *U* (*N_older_* = 12, *N_young_* = 12) = 33, *p* = 0.024. Young participants were also significantly more accurate (*M* = 98.55 ± 3.14) compared to older participants (*M* = 93.46 ± 6.28) when reporting the direction of new motion after exogenous reversals *U* (*N_older_* = 12, *N_young_* = 12) = 115, *p* = 0.01.

### 3.2. Alpha modulation in endogenous task

**Figure 4:**
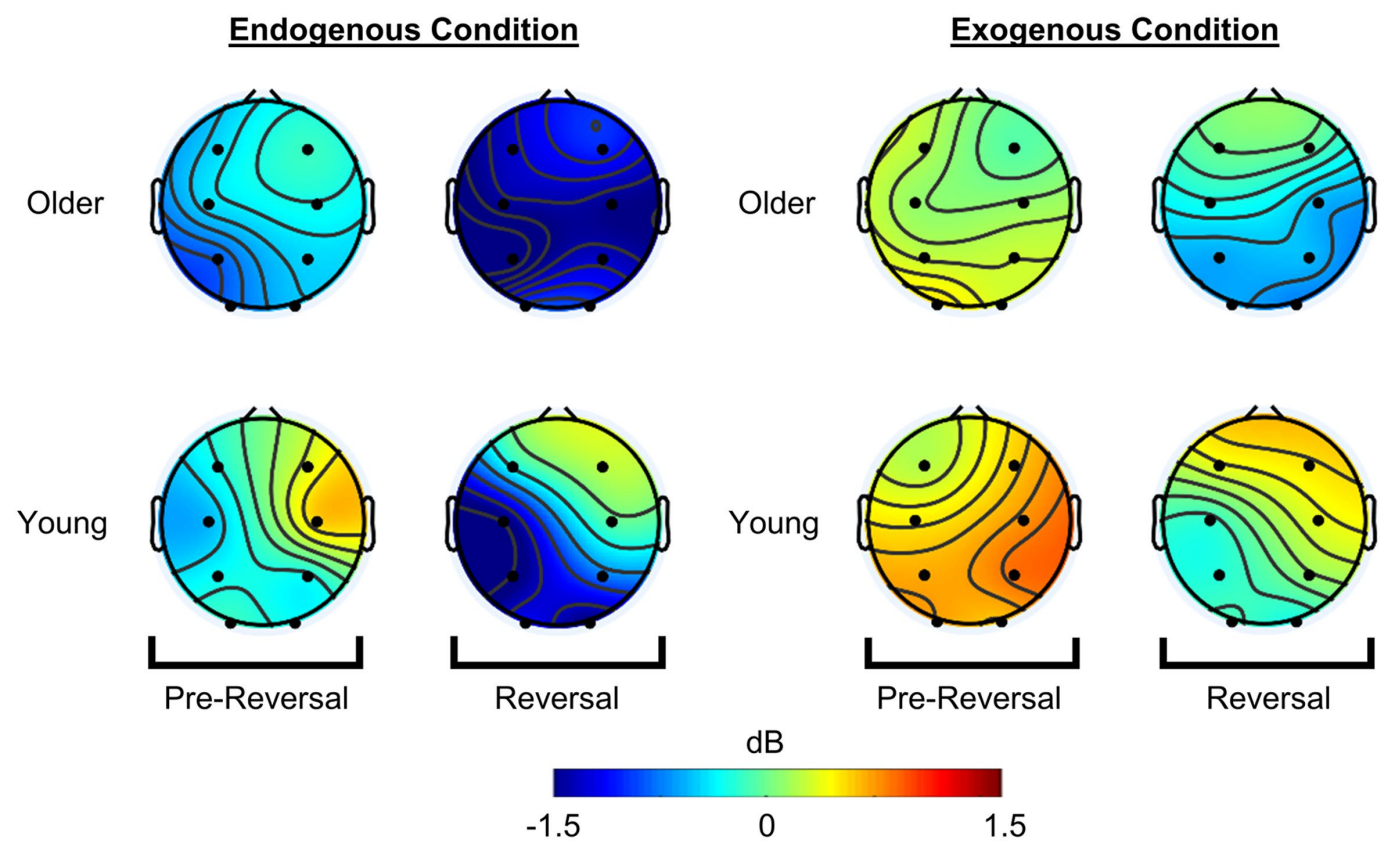
Reversal-related modulation of 11 Hz power. Colors indicate increase (red) or decrease (blue) of 11 Hz power during endogenous (left) and exogenous (right) conditions relative to activity in baseline window of non-reversal epochs. Reversal-related decrease in 11 Hz activity was confined in posterior areas for young adults (top) during endogenous reversals. Remarkably, this decrease was diffused throughout the cortex of older adults. 11 Hz activity was decreased at posterior locations for both groups during exogenous reversals.

Statistical parameters of main and interaction effects are shown in Table 2. Alpha modulation from pre-reversal to reversal window at the whole cortex is depicted in Figure 4.

**Table 2:** Summary of alpha modulation analyses (2 Group x 4 ROI x 2 Window) are shown for endogenous and exogenous conditions.

Factors (df)	Endogenous condition	Exogenous condition
Time (1, 22)	*F* = 20.270 ***p* < 0.001** *η_p_^2^* = 0.480	*F* = 6.762 ***p* = 0.016** *η_p_^2^* = 0.235
ROI (3, 66)	*F* = 2.924 *p* = 0.066	*F* = 0.485 *p* = 0.574
Group (1, 22)	*F* = 0.054 *p* = 0.819	*F* = 1.799 *p* = 0.194
Time × Group (1, 22)	*F* = 0.2 *p* = 0.887	*F* < 0.001 *p* = 0.986
ROI × Group (3, 60)	*F* = 1.601 *p* = 0.214	*F* = 0.170 *p* = 0.791
ROI × Time (3, 66)	*F* = 2.275 *p* = 0.108	*F* = 5.523 ***p* = 0.008** *η_p_^2^* = 0.201
ROI × Time x Group (3, 66)	*F* = 6.033 ***p* = 0.003** *η_p_^2^* = 0.215	*F* = 1.796 *p* = 0.865

*F* and *p* values are reported after Greenhouse–Geisser correction.

*F* = F-ratios, *p* = probabilities, *η*_p_^2^ = partial eta squared, df = degrees of freedom.

**Figure 5:**
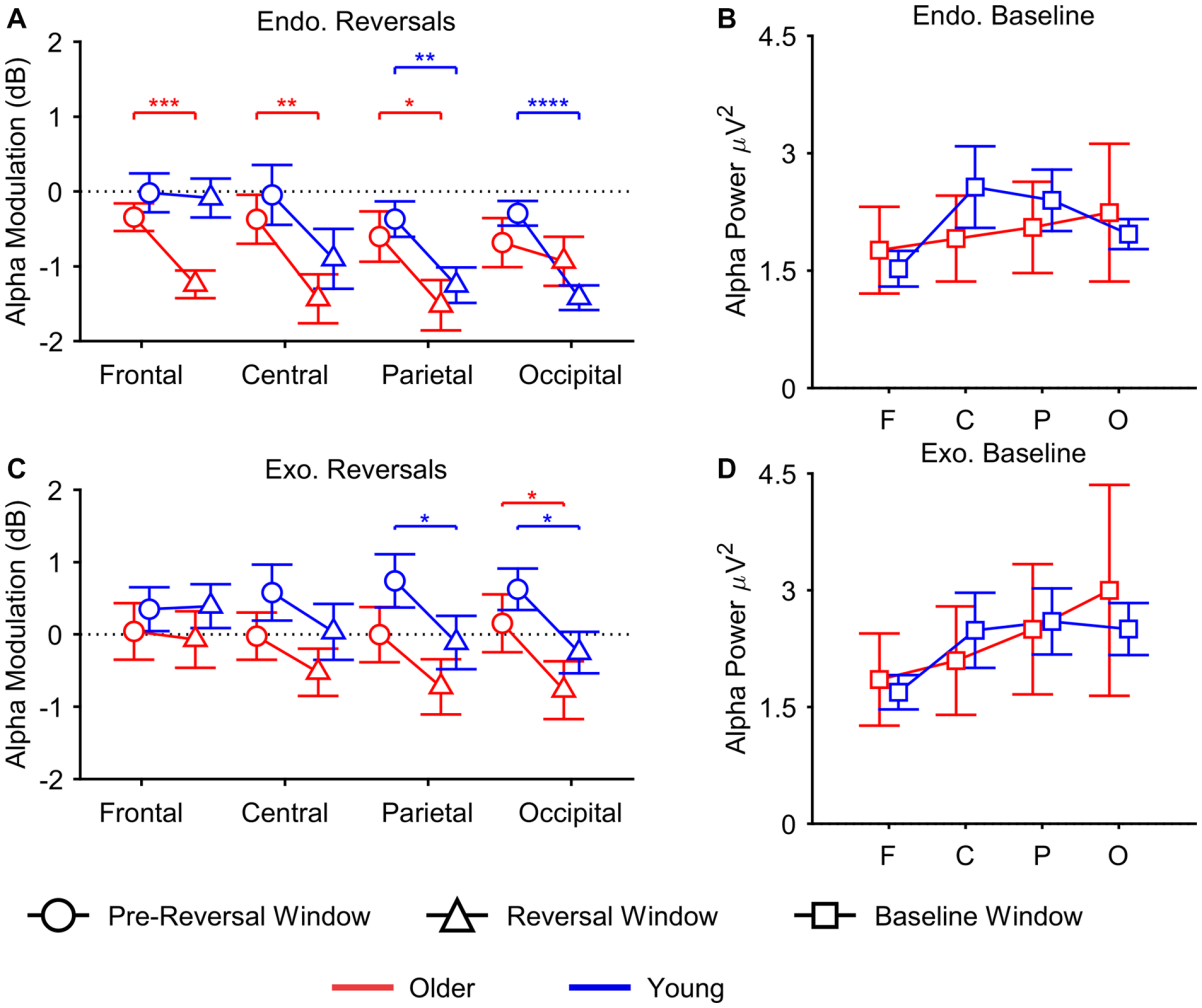
Visual depiction of EEG findings. **(A)** Young adults (blue line) had lower parietal and occipital alpha in reversal window (triangle: −350 to −50 ms) compared to pre-reversal window (circle: −950 to −650 ms) during endogenous (*Endo.*) reversals; whereas decrease occurred at frontal, central, and parietal locations in older group (red line). **(B)** Both groups showed similar levels of alpha activity in baseline window (square: −500 to 0 ms) of endogenous condition. **(C)** Alpha activity was lower during reversal (triangle: −250 to 50 ms) compared to pre-reversal window (circle: −850 to −550 ms) at parietal and occipital areas for young group during exogenous (*Exo.*) reversals. This decrease was observed at occipital area for older adults. **(D)** There were no group differences in alpha activity during baseline window of the exogenous condition. Asterisks are color matched with groups and show within group comparisons (*p < 0.05, **p < 0.01, ***p < 0.001, ****p < 0.0001). Error bars show %95 within subjects CI **(A,C)** and between subjects standard error of the mean **(B,D)**.

Overall, modulation of alpha power was not different between the groups (main effect: Group) or regions of interest (main effect: ROI). Alpha modulation was significantly lower in reversal compared to pre-reversal window (main effect: Time). A significant three-way interaction of Time × ROI × Group was found in the endogenous condition. Furthermore, linear trends of Time and ROI showed significant group differences in temporal modulation of alpha activity across different regions of interest *F* (1, 22) = 11.162, *p* = 0.003, *η_p_^2^* = 0.337. Specifically, reversal-related decrease was higher at posterior ROIs for young adults but it was higher at anterior ROIs for older adults. These results are in line with planned comparisons that showed significant pre-reversal to reversal decrease in alpha at frontal *t*(11) = 4.759, *p* = 0.001, Hedge’s *g_av_* = 0.54, central *t*(11) = 3.184, *p* = 0.009, Hedge’s *g_av_ = 0*.65, and parietal ROIs *t*(11) = 2.675, *p* = 0.022, Hedge’s *g_av_ = 0*.56 for older adults; and parietal *t*(11) = 3.650, *p* = 0.004, Hedge’s *g_av_* = 0.63 and occipital ROIs *t*(11) = 6.707, *p* = 0.000, Hedge’s *g_av_* = 1.31 for young adults (Figure 5). These results show that reversal-related alpha suppression was shifted towards anterior locations in older adults. Furthermore, regression analyses have shown that relative alpha power in reversal window predicts mean dwell times with frontal ROI for older and occipital ROI for young adults (Supplementary Figure S2), exclusively.

Baseline alpha power was not significantly different between the groups. However, it was significantly different between ROIs *F*(3, 66) = 3.503, *p* = 0.038, *η_p_^2^* = 0.137 (Figure 5). Post-hoc comparisons have shown that frontal baseline alpha power was significantly lower than parietal baseline alpha *t*(23) = 3.778, *p* = 0.001, Hedge’s *g_av_* = 0.36. Frontal alpha was also smaller compared to central and occipital ROIs, however, these comparisons did not reach significance after correcting for multiple comparisons (*p* > 0.004).

### 3.3. Alpha modulation in exogenous task

Alpha power in the reversal window was significantly lower than the activity in the pre-reversal window (main effect: Time; see Table 2). Furthermore, the magnitude of decrease in alpha was increased linearly from frontal to occipital areas (interaction: Time x ROI) as shown by linear trend contrasts of Time and ROI *F*(1, 22) = 7.715, *p* = 0.011, *η_p_^2^* = 0.260. However, this contrast did not reach significance after Bonferroni correction (*p* > 0.004). Planned comparisons have shown significant parietal *t*(11) = 2.267, *p* = 0.045, Hedge’s *g_av_* = 0.64 and occipital *t*(11) = 2.293, *p* = 0.012, Hedge’s *g_av_* = 0.63 decrease in alpha activity for young adults and significant decrease at occipital ROI for older adults *t*(11) = 2.268, *p* = 0.044, Hedge’s *g_av_* = 0.74 (Figure 5).

Baseline alpha activity was not different between the groups. There was a marginally significant effect of ROI on baseline alpha activity *F*(3, 66) = 3.583, *p* = 0.049, *η_p_^2^* = 0.140 (Figure 5). Post-hoc comparisons showed that frontal baseline activity was smaller than parietal baseline activity *t*(23) = 3.428, *p* = 0.002, Hedge’s *g_av_* = 0.39. The rest of the comparisons did not reach significance after correction (*p* > 0.004).

## 4. Discussion

### 4.1. Behavioral performance

Healthy aging is related to decreased cognitive performance in several domains. Performance impairments are usually reported in the form of slowed reaction times (Salthouse, 1996; Schmiedt-Fehr et al., 2011) and degree of slowing increases as task difficulty increases (Grady, 1998; Anstey and Smith, 1999; Schmiedt-Fehr et al., 2009). Aging also decreases perceptual reversals (Jalavisto, 1964) even when eye movements and pupil size are controlled for Aydin et al. (2013) or multistable stimuli are auditory (Kondo and Kochiyama, 2018). Our findings have replicated the age-related decrease of perceptual reversals. Aging was also shown to slow the reaction times to stimulus triggered reversals in the exogenous task, replicating the general slowing of reaction times in aging.

It may be argued that age-related decrease in reversal rates is secondary to age-related slowing and decreased accuracy rates. However, post-hoc correlation analyses did not show such relationship between reversal rates, reaction times, and omissions (see Supplementary Table S1). It may also be argued that older participants did not understood the task and hesitated to press buttons during endogenous task. However, this assumption is not plausible for two reasons: (i) it was made sure that all participants have seen both perceptual interpretations and - by using a control stimulus - associated correct responses to each percept before experiment begins and (ii) accuracy rates during the control task were well above %80 for older group, eliminating the possibility of task not being understood by older adults. Therefore, we argue that the age difference in reversal rates stems from age-related changes in perceptual processing and not other changes such as age-related slowing.

### 4.2. Alpha activity during endogenous reversals

Alpha responses are involved in sensory, motor, and cognitive processes (Başar et al., 1997; Klimesch et al., 1998; Başar et al., 2000). Its sensory function is operationalized as decreased activity in modality specific area following a simple sensory stimulation (Niedermeyer, 1993; Başar and Schürrmann, 1996). Another well-established finding is the decrease in alpha activity during and/or in anticipation to various cognitive tasks (Klimesch, 1997; Babiloni et al., 2006). As Başar et al. (1997) and many other researchers pointed out, there is no single and general pattern of alpha activity that correlates with all the different cognitive processes (Klimesch, 1999; Başar-Eroglu et al., 2016). Instead, there are distinct and distributed alpha networks that respond to specific events in different alpha sub-bands (Klimesch, 1999; İşoǧlu-Alkaç and Strüber, 2006). Here, we aimed to identify the age-related changes in alpha activity which is thought to be related to perceptual maintenance and destabilization of multistable percepts.

Alpha activity displays a reliable pattern in response to perceptual reversals in multistable perception tasks. Studies conducted on healthy young adults have repeatedly shown gradual decrease in alpha activity at posterior areas that starts approximately 1 s before the motor response that indicate the reversals (İşoğlu-Alkaç et al., 2000; Strüber and Herrmann, 2002; Başar-Eroglu et al., 2016). This was considered to represent the destabilization of activity in neural populations due to adaptation. This interpretation is rooted in findings of studies with different multistable stimuli (İşoğlu-Alkaç et al., 2000), data analysis methods (Mathes et al., 2010), and brain imaging techniques (Strüber and Herrmann, 2002). Our findings replicated the gradual decrease in parieto-occipital alpha activity during endogenous reversals in young adults. However, older adults showed a topographically distinct alpha response; occipital alpha response was diminished and instead it shifted to frontal, central, and parietal areas. Furthermore, this was reflected in regression analyses such that mean dwell times were predicted by frontal alpha in older and occipital alpha in young adults (Supplementary Figure S2). These supported our hypotheses regarding the diminished occipital response and compensatory alpha activity in older adults. Analysis of baseline alpha amplitudes did not show between-group differences in alpha activity that could have affected this topographical shift. Therefore, these findings indicate an age-related change in task driven alpha responses that are independent of baseline alpha activity.

A similar age-related shift in alpha topography was reported in previous literature: Kolev et al. (2002) employed a simple visual stimulation task and reported increased alpha (7–15 Hz) amplitude and phase-locking at anterior areas of middle aged adults (mean age = 53.6 ± 2.2) compared to younger adults. Furthermore, amplitude and phase-locking at occipital location were decreased. Similar findings were obtained for auditory stimulation, anterior alpha enhancement and phase-locking was increased in middle aged adults (Yordanova et al., 1998). Kolev et al. (2002) argued that sensory information within older brains is transferred to associative areas to compensate for reduced functionality of sensory brain areas. These studies show the same topographical pattern observed in this study even though the signal decomposition and data analysis methods (e.g., digital filtering, root mean squared-enhancement factor, phase-locking) are different than the ones applied in this study (e.g., Morlet wavelet convolution, baseline normalized time-frequency power). This indicates to a general age-related cortical reorganization of alpha networks in sensory and perceptual processing.

Studies on different animal species also show age-related functional decrement of neurons in the visual cortex. Signal to noise ratio of old V1 cells are decreased in rats (Ding et al., 2017), cats (Hua et al., 2006) and monkeys (Schmolesky et al., 2000; Yang et al., 2009). Shared conclusion of these studies was that aging decreased stimulus selectivity of older neurons due to reduced ability to retrieve sensory signals because of the noisy ongoing activity. In fact, human perceptual discrimination studies support this interpretation. A simple perceptual decision making study reported age-related slowing of reaction times both in visual and auditory modalities (Laurienti et al., 2006). Another study showed decreased perceptual discrimination accuracy in older adults, again, both in auditory and visual modalities (O'Brien et al., 2020). Literature indicates that decreased efficacy of sensory areas prolong or, with increased task demands, limit the perceptual discrimination capabilities in older animals, including humans. It appears that whole brain networks compensate for this inefficacy by recruiting additional alpha networks across the cortex.

Current findings might hint to a reason that explains decreased reversal rates in older adults. Traditionally, reversals thought to occur when the adaptation of percept-specific neural populations reach a certain threshold (Kohler, 1940; Orbach et al., 1963; Leopold and Logothetis, 1999; Kornmeier and Bach, 2012). As mentioned before, studies have considered reversal-related alpha response to be an index of adaptation process. This means that recruitment of additional alpha networks increases the number of neural populations that need to reach the adaptation threshold before a reversal can occur. Therefore, neural adaptation will take longer for older adults because compensatory alpha recruitment increases the number of neural populations to be activated. We argue that increased number of active alpha networks might have prolonged the adaptation process and dwell times in older adults, resulting in lower reversal rates.

### 4.3. Alpha activity during exogenous reversals

The most apparent difference in alpha response between exogenous and endogenous reversals is the time course of activity: decrease in former is steeper and shorter compared to the latter (Strüber and Herrmann, 2002; Mathes et al., 2010). Strüber and Herrmann (2002) argued that alpha activity preceding exogenous reversals is primarily a stimulus-driven response. This interpretation was in line with the results of Başar-Eroglu et al. (2016) where the difference in alpha response between healthy controls and schizophrenia patients was apparent during endogenous but not during exogenous reversals. In line, no impairment of stimulus-driven processing itself was expected in patients, but an impairment of *internal* processing of perceptual information maintaining and switching between endogenous stimulus perceptions. Similarly, current results displayed no difference in alpha response during exogenous reversals for the aging brain. However, aging is known to change topography of sensory alpha response (Yordanova et al., 1998; Kolev et al., 2002), which seem to contradict with the current results for the exogenous stimulus change. Schmiedt-Fehr et al. (2009) also dealt with a similar contradiction in a Go/NoGo paradigm and argued that involvement of higher-order processes might have improved the sensory function of alpha networks. We could argue that top-down responses to exogenous reversals in older adults might have eliminated the need for compensatory alpha activation. Future studies should investigate the effect of varying degrees of stimulus strength on top-down activation and its relationship to compensatory alpha responses to further explore this issue. Alternatively, choosing 11 Hz as the center frequency and not dividing alpha into sub-bands might have masked the group differences in anterior alpha responses in the current study.

### 4.4. Limitations

There was an unequal number of males and females within the sample. Therefore, our findings and interpretations should be evaluated under this constraint. Furthermore, aging and its effects on functional neural activity is a slow and gradual process. This study investigated young adults and older adults exclusively. However, including middle aged and older adults with even more advanced ages (+80 years of age) could have shed light into the gradual effects of aging on brain oscillations. Future studies should consider the limitation of severely decreased reversal rates after 80 years of age and design experiments accordingly.

Previous studies reported a shift in the alpha frequency peak with advancing age. There are also studies that specified that low and mid alpha ranges are the most involved during multistable perception. We have not used individual alpha frequencies or analyzed sub-bands of alpha activity in this study. Furthermore, we argue that this could have been a reason for the lack of between-group difference in the exogenous task. Future studies should delineate the effect of individual alpha frequencies and sub-bands of alpha band to examine if they alter age-related differences in endogenous and exogenous tasks.

Multistable perception requires the collaboration of top-down and bottom-up processes which are represented in multiple frequency bands across the selectively distributed oscillatory networks. Therefore, investigating the interaction of multiple frequency bands can provide important insights that cannot be captured by analysis of a single oscillatory activity.

## 5. Conclusion

Topographical change in reversal related alpha responses indicated functional deterioration of sensory areas and recruitment of compensatory alpha networks to facilitate perceptual maintenance in older adults. This indicated that reversals were driven predominantly by bottom-up processes in the older group. Recruitment of additional alpha networks in the older group might have prolonged the satiation of percepts due to larger cortical representation. This change might have led to decreased reversal rates. The age groups showed comparable alpha responses in the control condition. This task-specific compensatory activation of alpha networks indicates a deficit of the occipital cortex of elderly that disrupts the extraction and maintenance of multiple perceptual representations from an unchanging visual input. This study provided another evidence for the age-related compensatory activation patterns of selectively distributed oscillatory networks.

## Data availability statement

The raw data supporting the conclusions of this article will be made available by the authors, without undue reservation.

## Ethics statement

The studies involving human participants were reviewed and approved by Bremen University. The patients/participants provided their written informed consent to participate in this study.

## Author contributions

AW, BM, and CB-E contributed to the conception and design of the study. KK analyzed the EEG data, conducted the statistical analyses, and wrote the manuscript. All authors contributed to the article and approved the submitted version.

## Funding

This study (project number 119K411) was supported by the Turkish National Science and Research Council (TÜBİTAK) during data analysis. Open access publication fee was funded University of Bremen.

## Conflict of interest

The authors declare that the research was conducted in the absence of any commercial or financial relationships that could be construed as a potential conflict of interest.

## Publisher’s note

All claims expressed in this article are solely those of the authors and do not necessarily represent those of their affiliated organizations, or those of the publisher, the editors and the reviewers. Any product that may be evaluated in this article, or claim that may be made by its manufacturer, is not guaranteed or endorsed by the publisher.

## Supplementary material

SUPPLEMENTARY VIDEO S1 Video of stroboscopic alternative motion stimulus in endogenous condition.

SUPPLEMENTARY VIDEO S2 Video of control stimulus (i.e. exogenous stimulus) in exogenous condition.

SUPPLEMENTARY FIGURE S1 Characteristics of the wavelet used in time-frequency decomposition. (top) Frequency domain representation of the wavelet, (middle) gaussian used to produce the Morlet wavelet, (bottom) time-domain representation of the Morlet wavelet.

SUPPLEMENTARY FIGURE S2 Visual depiction of regression analyses. Scatterplots of alpha modulation (dB) and log-transformed dwell times are shown for frontal (*left, denoted as* F) and occipital (*right, denoted as* O) areas. Frontal alpha response significantly predicted dwell times only for older (red) adults *F* (1, 10) = 5.557, *p* = 0.04. On the contrary, occipital alpha response was a significant predictor of dwell times for young (blue) adults *F* (1, 10) = 25.250, *p* < 0.001, but not for older adults. R2 values are color matched with group colors of older and young adults. Asterisks indicate significant regression models (**p* <0 .05, ****p* < 0.001). Mean dwell times of vertical and horizontal percepts were averaged and transformed to base 10 logarithm before analyses.

SUPPLEMENTARY TABLE S1 SPSS output of Spearman Rho correlations between (i) reversal rates and omissions, and (ii) reversal rates and reaction times. There were no significant correlations between these behavioral measures.
