# Supplementary figures and images for: Multistable perception elicits compensatory alpha activity in older adults
Source: Front Aging Neurosci. 2023 May 25;15:1136124. doi: 10.3389/fnagi.2023.1136124 (PMC10249475; doi:10.3389/fnagi.2023.1136124)

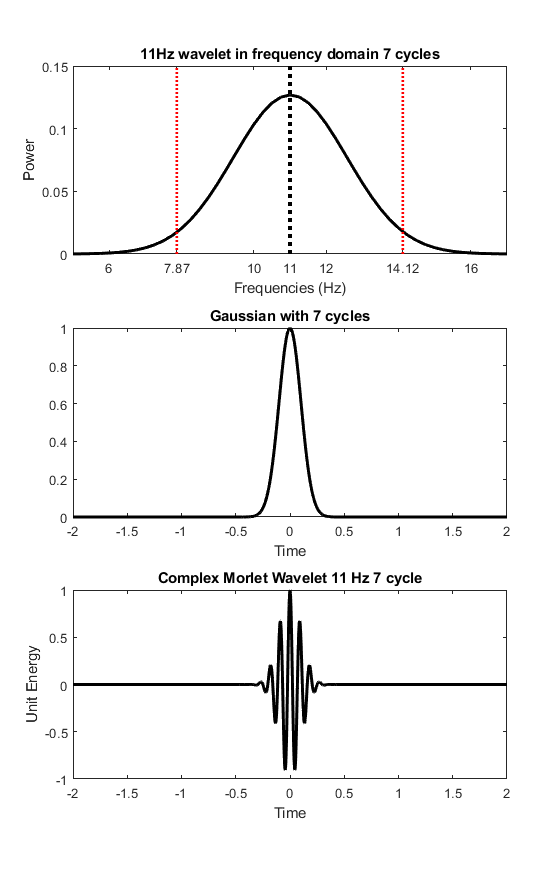

Supplement: SUPPLEMENTARY FIGURE S1 — Characteristics of the wavelet used in time-frequency decomposition. (top) Frequency domain representation of the wavelet, (middle) gaussian used to produce the Morlet wavelet, (bottom) time-domain representation of the Morlet wavelet. [file Image_1.tif]

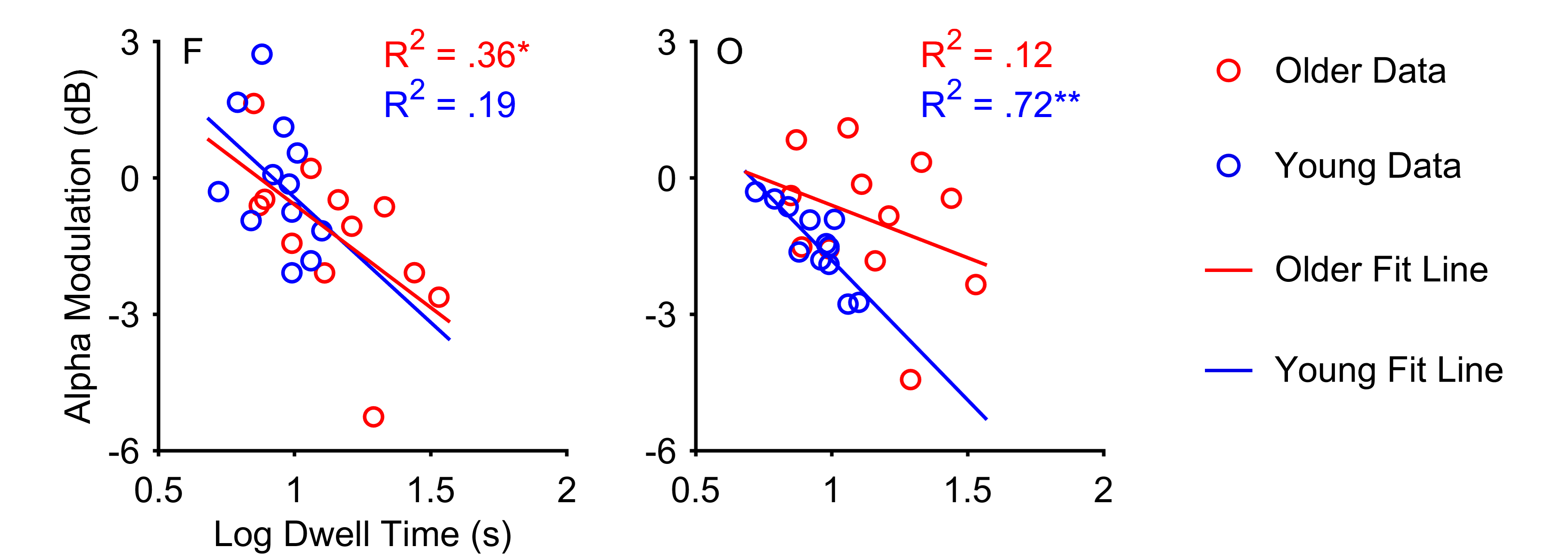

Supplement: SUPPLEMENTARY FIGURE S2 — Visual depiction of regression analyses. Scatterplots of alpha modulation (dB) and log-transformed dwell times are shown for frontal (left, denoted as F) and occipital (right, denoted as O) areas. Frontal alpha response significantly predicted dwell times only for older (red) adults F (1, 10) = 5.557, p = 0.04. On the contrary, occipital alpha response was a significant predictor of dwell times for young (blue) adults F (1, 10) = 25.250, p < 0.001, but not for older adults. R2 values are color matched with group colors of older and young adults. Asterisks indicate significant regression models (*p <0 .05, ***p < 0.001). Mean dwell times of vertical and horizontal percepts were averaged and transformed to base 10 logarithm before analyses. [file Image_2.tif]
