# Supplementary material for: Multistable perception elicits compensatory alpha activity in older adults
Source: Front Aging Neurosci. 2023 May 25;15:1136124. doi: 10.3389/fnagi.2023.1136124 (PMC10249475; doi:10.3389/fnagi.2023.1136124)
Supplement: SUPPLEMENTARY TABLE S1 — SPSS output of Spearman Rho correlations between (i) reversal rates and omissions, and (ii) reversal rates and reaction times. There were no significant correlations between these behavioral measures. [file Table_1.PDF]

```
DATASET ACTIVATE DataSet1.

SAVE OUTFILE='E:\Backups\Matlab '+
    'Directory\2020_MSP_EEG_Scrpt-Data_Mert\2020_MSPAging_EEG_Mert\Data_MertVe
rsions\Data_Analysis\'+
    'Mert_AlphaArticle\Alpha_Birgit\spss_data\alpha_behavioral.sav'
  /COMPRESSED.
NONPAR CORR
  /VARIABLES=reversalRate omissions
  /PRINT=SPEARMAN TWOTAIL NOSIG
  /MISSING=PAIRWISE.
```

Correlation Between Omissions and Reversal Rates by Groups

Notes

Output Created		25-APR-2023 20:19:29
Comments		
Input	Data	E:\Backups\Matlab Directory\2020_MSP_EEG_Scrpt-Data_Mert\2020_MSPAging_EEG_Mert\Data_MertVersions\Data_Analysis\Mert_AlphaArticle\Alpha_Birgit\spss_data\alpha_behavioral.sav
	Active Dataset	DataSet1
	Filter	usedInAnalysis=1 (FILTER)
	Weight	<none>
	Split File	Group
	N of Rows in Working Data File	24
Missing Value Handling	Definition of Missing	User-defined missing values are treated as missing.
	Cases Used	Statistics for each pair of variables are based on all the cases with valid data for that pair.

Notes

Syntax		NONPAR CORR<br><br>/VARIABLES=reversalRate omissions<br>/PRINT=SPEARMAN TWOTAIL NOSIG<br>/MISSING=PAIRWISE.
Resources	Processor Time	00:00:00.00
	Elapsed Time	00:00:00.00
	Number of Cases Allowed	629145 cases[a]

a. Based on availability of workspace memory

Group = older

Correlations[a]

			reversalRate	omissions
Spearman's rho	reversalRate	Correlation Coefficient	1.000	.190
		Sig. (2-tailed)	.	.555
		N	12	12
	omissions	Correlation Coefficient	.190	1.000
		Sig. (2-tailed)	.555	.
		N	12	12

a. Group = older

Group = younger

Correlations[a]

			reversalRate	omissions
Spearman's rho	reversalRate	Correlation Coefficient	1.000	-.459
		Sig. (2-tailed)	.	.134
		N	12	12
	omissions	Correlation Coefficient	-.459	1.000
		Sig. (2-tailed)	.134	.
		N	12	12

a. Group = younger

```
SPLIT FILE OFF.
NONPAR CORR
  /VARIABLES=reversalRate omissions
  /PRINT=SPEARMAN TWOTAIL NOSIG
  /MISSING=PAIRWISE.
```

Correlation Between Omissions and Reversal Rates (ALL PARTICIPANTS)

Notes

Output Created		25-APR-2023 20:20:30
Comments		
Input	Data	E:\Backups\Matlab Directory\2020_MSP_EEG_Scrpt-Data_Mert\2020_MSPAging_EEG_Mert\Data_MertVersions\Data_Analysis\Mert_AlphaArticle\Alpha_Birgit\spss_data\alpha_behavioral.sav
	Active Dataset	DataSet1
	Filter	usedInAnalysis=1 (FILTER)
	Weight	<none>
	Split File	<none>
	N of Rows in Working Data File	24
Missing Value Handling	Definition of Missing	User-defined missing values are treated as missing.
	Cases Used	Statistics for each pair of variables are based on all the cases with valid data for that pair.
Syntax		NONPAR CORR /VARIABLES=reversalRate omissions /PRINT=SPEARMAN TWOTAIL NOSIG /MISSING=PAIRWISE.

Notes

Resources	Processor Time	00:00:00.00
	Elapsed Time	00:00:00.00
	Number of Cases Allowed	629145 cases[a]

a. Based on availability of workspace memory

Correlations

			reversalRate	omissions
Spearman's rho	reversalRate	Correlation Coefficient	1.000	-.342
		Sig. (2-tailed)	.	.102
		N	24	24
	omissions	Correlation Coefficient	-.342	1.000
		Sig. (2-tailed)	.102	.
		N	24	24

```
NONPAR CORR
  /VARIABLES=reversalRate MeanRT
  /PRINT=SPEARMAN TWOTAIL NOSIG
  /MISSING=PAIRWISE.
```

Correlation Between Reaction Time and Reversal Rates (ALL PARTICIPANTS)

Notes

Output Created		25-APR-2023 20:21:13
Comments		
Input	Data	E:\Backups\Matlab Directory\2020_MSP_EEG_Scrpt-Data_Mert\2020_MSPAging_EEG_Mert\Data_MertVersions\Data_Analysis\Mert_AlphaArticle\Alpha_Birgit\spss_data\alpha_behavioral.sav
	Active Dataset	DataSet1
	Filter	usedInAnalysis=1 (FILTER)
	Weight	<none>
	Split File	<none>
	N of Rows in Working Data File	24
Missing Value Handling	Definition of Missing	User-defined missing values are treated as missing.
	Cases Used	Statistics for each pair of variables are based on all the cases with valid data for that pair.
Syntax		NONPAR CORR /VARIABLES=reversalRate MeanRT /PRINT=SPEARMAN TWOTAIL NOSIG /MISSING=PAIRWISE.
Resources	Processor Time	00:00:00.02
	Elapsed Time	00:00:00.00
	Number of Cases Allowed	629145 cases[a]

a. Based on availability of workspace memory

Correlations

			reversalRate	MeanRT
Spearman's rho	reversalRate	Correlation Coefficient	1.000	-.250
		Sig. (2-tailed)	.	.238
		N	24	24
	MeanRT	Correlation Coefficient	-.250	1.000
		Sig. (2-tailed)	.238	.
		N	24	24
